# An Unusual Etiology of Bayés' Syndrome: Fabry Disease

**DOI:** 10.1111/anec.70220

**Published:** 2026-07-01

**Authors:** Nicholas E. Kunce, Andre Silva, Mark Haigney, Adam Kisling

**Affiliations:** ^1^ Department of Medicine, Division of Cardiology Brooke Army Medical Center Fort Sam Houston Texas USA; ^2^ Uniformed Services University of the Health Sciences Bethesda Maryland USA

**Keywords:** atrial fibrillation, Bayés' syndrome, Fabry disease, infiltrative cardiomyopathy, interatrial block, lysosomal storage disorder

## Abstract

We present the case of a 72‐year‐old female with Fabry disease who developed an advanced interatrial conduction block and atrial fibrillation, a condition known as Bayés' syndrome. Fabry disease is an X‐linked inherited lysosomal storage disorder that results in the accumulation of globotriaosylceramide in various organs, including the heart. While this condition has been shown to cause cardiac conduction disease, Bayés' syndrome as a result of Fabry disease has not been reported in the literature. This case report suggests that Fabry disease may precipitate atrial cardiomyocyte damage at Bachmann's bundle leading to interatrial block and supraventricular arrhythmia.

## Graphical abstract

This patient with gene confirmed Fabry disease had advanced interatrial conduction block as seen on atrial‐specific vector tracing demonstrating sinus bradycardia with first‐degree AV block and sinus P‐wave terminal delay with biphasic morphology. The patient was later diagnosed with atrial fibrillation, which led to the diagnosis of Bayés' syndrome.

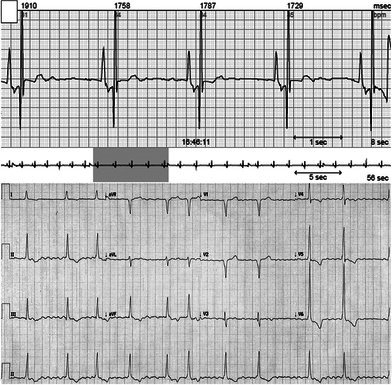

## 1 Introduction

Fabry disease, an X‐linked recessive lysosomal storage disorder, stems from pathologic variants within the GLA gene, which codes for the enzyme α‐galactosidase A. This enzyme deficiency results in the buildup of globotriaosylceramide (Gb3) and associated glycosphingolipids which subsequently leads to multi‐organ expressions of the disease over time. Although it primarily affects males, Fabry disease can affect females on a spectrum due to random X‐chromosomal inactivation. Although the primary effects of Fabry disease on the heart are ventricular hypertrophy, diastolic dysfunction, and aortic root dilation, Gb3 accumulation in cardiomyocytes and cardiac conduction tissue can lead to arrhythmias and conduction disease (Germain 2010; Namdar 2016). The manifestations of these electrophysiologic changes have previously been described to include bradycardia, PQ‐interval shortening, atrioventricular (AV) block, repolarization abnormalities, atrial fibrillation, and ventricular tachycardia (Namdar 2016). However, advanced interatrial block (IAB) in patients with Fabry disease has not previously been reported. The association of advanced IAB and supraventricular arrhythmia is called Bayés' syndrome (Baranchuk et al. 2018). The eponym was formally proposed by Conde and Baranchuk (2014), in recognition of the foundational work of Bayés de Luna and colleagues, and has since been broadly adopted in the cardiology literature. Importantly, Bayés' syndrome has been increasingly recognized as a contributor to unexplained transient ischemic attacks and cryptogenic ischemic stroke, and should be considered and investigated as a potential cause of paroxysmal atrial fibrillation (Arboix et al. 2017). In this report, we describe a case where Bayés' syndrome was diagnosed in a female patient with Fabry disease.

## 2 Case Report

A 72‐year‐old female with known Fabry disease presented to our cardiology clinic for evaluation of cardiac involvement after a recent episode of palpitations. The episode was brief, resolved spontaneously, and was not associated with other symptoms such as chest pain, dyspnea, lightheadedness, or syncope. She was otherwise asymptomatic and had a normal cardiovascular physical exam.

She had been clinically diagnosed with Fabry disease in her early 40s after an ophthalmologic evaluation revealed whorls in her cornea. She was considered a carrier of Fabry disease at that time. A few years before our evaluation, she underwent genetic testing confirming heterozygosity for a pathogenic variant in the GLA gene (c.718_719delAA, p.K240EfsX9), with a leukocyte α‐galactosidase A enzyme activity of 17 nmol/h/mg protein (reference range 20.3–60.9 nmol/h/mg protein), consistent with a diagnosis of Fabry disease. Over the past decade, she had been evaluated for rare occurrences of palpitations that had prompted further workup in the setting of her Fabry disease. A transthoracic echocardiogram 3 years before our evaluation found concentric left ventricular hypertrophy (1.4 cm) with normal left ventricular systolic function and grade 3 diastolic dysfunction. Cardiac magnetic resonance imaging (cMRI) 1 year before our evaluation revealed a mildly dilated ascending aorta (4.1 cm) and asymmetric septal hypertrophy (1.2 cm) without abnormal late gadolinium enhancement (LGE) in the left ventricular myocardium. Finally, 1 year prior to our evaluation, an ambulatory heart rhythm monitor (AHRM) revealed brief runs of asymptomatic paroxysmal supraventricular tachycardia. Before our evaluation, the patient had declined enzyme replacement therapy (ERT) for Fabry disease due to a desire to avoid frequent infusions.

Upon presentation to our clinic, she underwent evaluation with an ECG, an AHRM, and a cMRI. Her 12‐lead electrocardiogram (ECG) at that time was notable for P‐wave prolongation of greater than 120 milliseconds (ms) measured in V1 and notched (bifid) P‐waves consistent with partial IAB, without a final negative P‐wave deflection in the inferior leads to suggest typical or atypical advanced IAB (Figure 1). The 7‐day AHRM was a Carnation Ambulatory Monitor (CAM), and it demonstrated predominantly sinus bradycardia with first‐degree AV block but also revealed sinus P‐wave terminal delay with biphasic morphology consistent with advanced IAB (Figure 2A), 14 episodes of non‐sustained atrial tachycardia, and rare premature atrial contractions (PACs) which demonstrated fragmented P‐waves (Figure 2B). The cMRI demonstrated stable myocardial wall thickening and normal‐sized atria but also demonstrated new patchy mid‐myocardial delayed LGE within the basal lateral wall of the left ventricle, which was concerning for progression of her Fabry disease.

**FIGURE 1:**
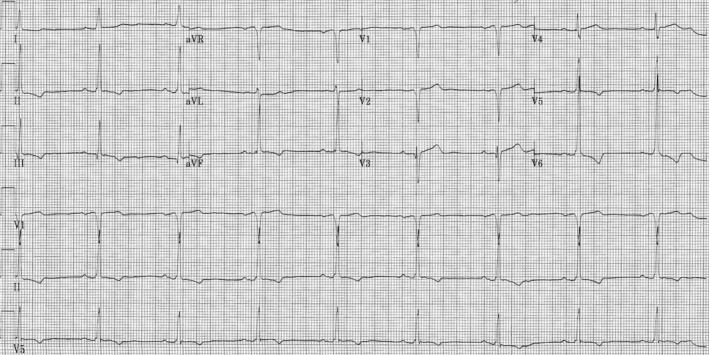
A 12‐lead ECG demonstrating sinus bradycardia and P‐wave prolongation of at least 120 ms measured in V1 and the inferior leads. The P‐waves are notched (bifid) in a pattern that is more consistent with IAB than left atrial enlargement. The P‐waves do not show a final negative deflection in the inferior leads, and therefore do not meet criteria for typical or atypical advanced IAB; the surface ECG is most consistent with partial IAB. There is left ventricular hypertrophy by Sokolow‐Lyon criteria with ST segment and T wave changes consistent with repolarization abnormalities in the setting of Fabry disease, but there is an absence of other findings of Fabry disease such as PQ‐interval shortening.

**FIGURE 2:**
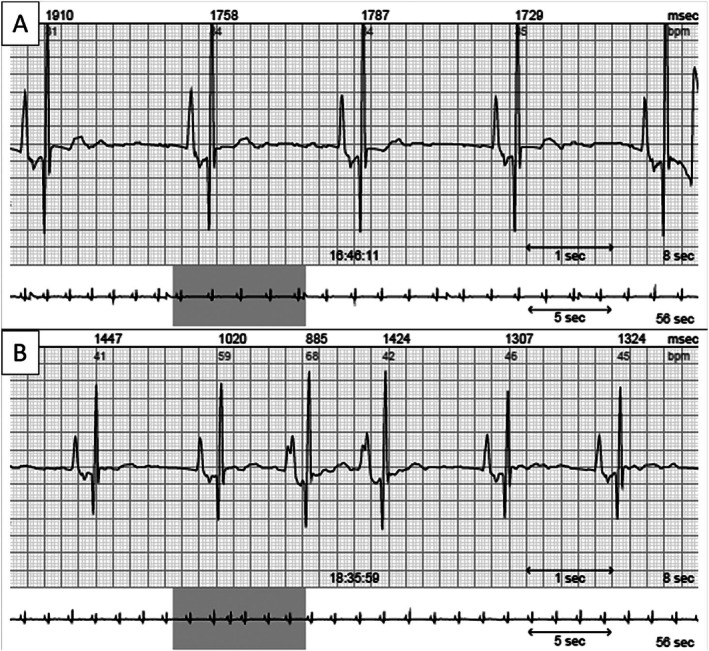
AHRM approximating a Lewis lead vector. (A) Sinus bradycardia with first‐degree AV block and sinus P‐wave terminal delay with biphasic morphology, consistent with advanced IAB unmasked by P‐wave‐centric ambulatory monitoring. (B) Sinus bradycardia with first‐degree AV block and a pair of PACs with wide, fragmented P‐waves.

Laboratory studies obtained at the time of her initial cardiology evaluation were notable for a hemoglobin of 13.2 g/dL, white blood cell count of 5.7 × 10^3^/μL, platelet count of 242 × 10^3^/μL, serum creatinine of 0.59 mg/dL, and an estimated glomerular filtration rate of 95 mL/min/1.73 m^2^. Inflammatory markers (ESR and CRP), natriuretic peptides, and cardiac troponin were not obtained, as the clinical presentation was not suggestive of acute ischemia, heart failure decompensation, or systemic inflammation.

Given the cardiac involvement demonstrated in these studies, the patient elected to pursue ERT with agalsidase beta infusions every 2 weeks. Approximately 7 months after initiation of ERT, she was re‐evaluated in our clinic and was found to be in asymptomatic atrial fibrillation with slow ventricular response (Figure 3). She was started on a direct oral anticoagulant. The development of atrial fibrillation was concerning for progression of her cardiac manifestations despite ERT, and a repeat cMRI was ordered. It showed patchy areas of delayed LGE throughout the myocardium with a focal area in the mid‐lateral left ventricular wall which had mildly progressed compared to her most recent cMRI. Atrial fibrosis could not be detected on the cMRI. The patient's left atrial size remained normal throughout her course.

**FIGURE 3:**
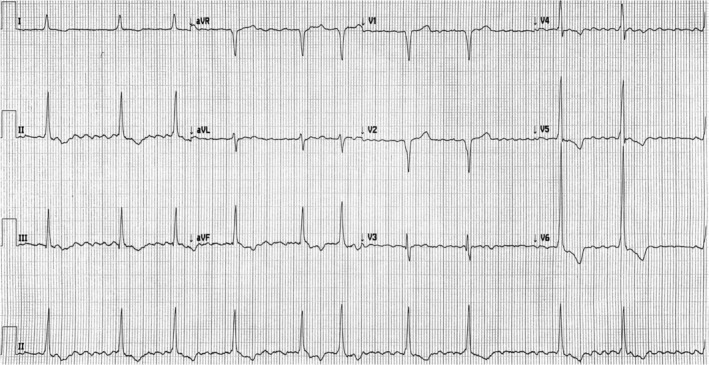
A 12‐lead ECG demonstrating atrial fibrillation with slow ventricular response and left ventricular hypertrophy by Sokolow‐Lyon criteria with associated repolarization abnormality.

## 3 Discussion

This case represents, to our knowledge, the first report of Bayés' syndrome in a patient with genetically confirmed Fabry disease. Several features of this case warrant emphasis at the outset. First, the patient's surface 12‐lead ECG demonstrated partial IAB rather than advanced IAB, but advanced IAB physiology was unmasked on a P‐wave‐centric ambulatory monitor approximating a Lewis lead vector. This illustrates an important diagnostic principle: advanced IAB may be present despite a surface 12‐lead ECG that meets criteria only for partial IAB, and high‐resolution atrial signal acquisition can be essential to establishing the diagnosis. Second, the patient subsequently developed atrial fibrillation despite initiation of enzyme replacement therapy, raising important questions about whether ERT modifies the natural history of atrial conduction disease in Fabry disease. Third, the recognition of Bayés' syndrome led to closer surveillance and earlier identification of asymptomatic atrial fibrillation, allowing initiation of anticoagulation prior to a clinically apparent cardioembolic event. The remainder of the discussion places these observations in the context of the published literature.

Interatrial block (IAB) is caused by delayed conduction between the right and left atrium due to impairment of Bachmann's bundle (BB). IAB has been described as having three patterns: first‐degree (P‐wave ≥ 120 ms in the absence of left atrial enlargement [LAE]), second‐degree (transient presence of first or third‐degree pattern), and third‐degree (P‐wave ≥ 120 ms ± biphasic morphology in the inferior leads), and second‐degree (transient presence of first or third‐degree pattern) (Bayés de Luna et al. 2012). In third‐degree, also called advanced IAB, the stimulus from the sinus node is completely blocked in the BB and retrograde activation of the left atrium (LA) occurs through an area in the vicinity of the coronary sinus (Baranchuk et al. 2018; Bayés de Luna et al. 2012). Patients with advanced IAB have been found to have a higher incidence of paroxysmal supraventricular tachyarrhythmias, particularly atrial fibrillation or atrial flutter, compared to control (Bayés de Luna et al. 1988).

The surface ECG presentations of LAE and IAB can be difficult to distinguish. Similar to IAB, classic LAE criteria include a broad, notched P wave of ≥ 110 ms duration in lead II. However, while advanced IAB presents with a biphasic morphology in the inferior leads, LAE presents with a biphasic morphology in lead V1 with the terminal negative deflection being at least 40 ms in duration and 1 mm in depth. When these two entities cannot be distinguished on ECG, cardiac imaging can be used to assess the presence of LAE. This patient was repeatedly assessed with echocardiography and cMRI and was consistently found to have normal left atrial size. In cases of heart failure secondary to infiltrative disorders, this distinction may become even more challenging. Fabry disease may lead to heart failure with chronically elevated left ventricular end‐diastolic pressure, which can in turn lead to LAE. In such a case, it could be especially difficult to determine if P‐wave prolongation was secondary to IAB, LAE, or a combination of the two.

In the case of our patient, her initial ECG demonstrated evidence of interatrial conduction block with P‐waves ≥ 120 ms in duration, particularly evident in V1 and the inferior leads. While she did not have markedly biphasic P‐waves in leads II, III, and aVF on surface ECG, she did have notched P‐waves illustrating the delay in conduction to the LA in the context of normal LA size (Figure 1). Bayés de Luna and colleagues have described atypical patterns of advanced IAB in which the classic biphasic morphology in all three inferior leads may be incomplete on the surface 12‐lead ECG, but each pattern still requires a final negative inflection in lead aVF reflecting retrograde left atrial activation (Bayés de Luna et al. 2018). Our patient's surface ECG did not demonstrate a clear final negative deflection in the inferior leads, and is therefore most accurately classified as partial IAB rather than typical or atypical advanced IAB. Furthermore, her ambulatory rhythm monitoring showed P‐wave prolongation as well as distinct biphasic morphology in the P‐waves consistent with advanced IAB (Figure 2). The discrepancy between the surface 12‐lead ECG and the P‐wave‐centric ambulatory monitor in this patient is itself a clinically important observation: advanced IAB physiology, with complete Bachmann's bundle blockade and retrograde left atrial activation, was demonstrated only with a recording vector specifically designed to optimize atrial signal acquisition. This finding suggests that surface 12‐lead ECG may underestimate the prevalence of advanced IAB, and that high‐resolution P‐wave‐centric monitoring can unmask the diagnosis in patients in whom only partial IAB is apparent on the standard surface ECG. A CAM was selected as the AHRM for this patient because it is a P‐wave centric AHRM which provides a more detailed assessment of atrial activity when compared to a traditional 12‐lead ECG because of its vector configuration, which is similar to that of a Lewis lead (Bardy Diagnostics Inc. 2024). Electrocardiography acquisition vectors designed specifically to provide detailed analysis of atrial signals offer a greater capability to diagnose conditions like advanced IAB and enhance our understanding of atrial conduction abnormalities (Rho et al. 2018; Smith et al. 2016). Because of these diagnostic characteristics, the P‐wave centric AHRM used in this case is sufficient for the diagnosis of advanced IAB and may have greater sensitivity for the condition than the traditional 12‐lead ECG based definition of advanced IAB. Given her history of paroxysmal supraventricular tachycardia, our patient was diagnosed with Bayés' syndrome at the point in time when she was found to have advanced IAB on her P‐wave‐centric ambulatory monitor.

The pathogenesis of IAB is not entirely clear. However, research has shown patients with IAB were consistently found to have intracellular damage and accumulation of collagen and glycogen between cells disturbing the structural arrangement of normal atrial muscle (Legato et al. 1974). We hypothesize that our patient's Bayés' syndrome was the result of her underlying Fabry disease, given what is known in the literature regarding Gb3 accumulation in cardiomyocytes and cardiac conduction tissue. We theorize that Gb3 accumulation caused damage to the BB and the surrounding atrial tissue and led to the electrophysiologic conditions that prompted the development of advanced IAB and therefore Bayés' syndrome.

Given our patient's age, it is also possible that there was a component of degenerative fibrosis of the atrial tissue which amplified the electrophysiologic effects of Gb3 accumulation. Elderly patients may develop advanced IAB because of age‐related atrial fibrosis, decreased cellular connectivity, and loss of atrial muscle mass. While the clinical presentation and imaging abnormalities in this case indicate Fabry disease as a primary contributor, age‐related factors could also have contributed to our patient's advanced IAB. Literature has supported that Bayés' syndrome can be illustrated on imaging techniques such as cMRI that show LGE consistent with atrial fibrosis, but atrial fibrosis was unable to be assessed on our patient's cMRI (Hernandez‐Betancor et al. 2017). However, as this is the first reported case of Bayés' syndrome believed to be caused by Fabry disease, it is difficult to know the sensitivity and specificity of imaging techniques to support the diagnosis given the spectrum of disease.

Although the patient had been receiving several months of ERT for her Fabry disease, it appears that this treatment did not effectively arrest the progression of the disease regarding the development of atrial fibrillation. Atrial fibrillation is associated with a variety of risk factors. In this case, the patient had advanced IAB and Fabry disease in addition to being elderly. While idiopathic atrial fibrillation is common in the elderly, atrial fibrillation is also strongly associated with advanced IAB and Fabry disease. Although the magnitude of increased risk of atrial fibrillation in patients with Fabry disease is not well known, there is a correlation between Fabry disease and arrhythmias, especially when there is increased ventricular wall thickness, atrial dilation, and diastolic dysfunction (Namdar 2016). For this reason, it is difficult to determine with confidence the histologic and physiologic factors that contributed most strongly to the development of atrial fibrillation in this case. Regardless, the clinical implication of Bayés' syndrome is that advanced IAB remains a strong risk factor for developing atrial arrhythmias like atrial fibrillation and thus, cardioembolic events. Fortunately, in this case, the patient was followed up more frequently after the diagnosis of Bayés' syndrome which resulted in her asymptomatic atrial fibrillation being identified early. As a result, she was started on anticoagulation before the occurrence of a cardioembolic event. This implication continues to raise the question of whether early anticoagulation in patients with advanced IAB has utility in the prevention of cardioembolic events, but further studies are required.

The presence of Bayés' syndrome in this patient with Fabry disease raises concern for the development of advanced IAB and atrial fibrillation in other infiltrative cardiomyopathies by a similar mechanism. While atrial fibrillation and advanced IAB have been associated with cardiac amyloidosis (Lindow and Lindqvist 2021), no associations between Bayés' syndrome and other infiltrative cardiomyopathies such as cardiac sarcoidosis, hemochromatosis, Pompe disease, or Danon disease have been described to our knowledge. This may indicate either that amyloidosis and Fabry disease have a unique effect on cardiac conduction or more likely that Bayés' syndrome is an underrecognized sequela of infiltrative cardiomyopathies. These issues will require further investigation and increased provider awareness of Bayés' syndrome as a clinical entity.

The cerebrovascular implications of this case warrant additional consideration. Although small‐vessel disease accounts for the majority of lacunar infarcts, less than 5% are attributable to unusual etiologies, including hematologic disorders, infectious or inflammatory arteritides, and Fabry disease, and establishing the correct etiologic diagnosis is essential, as therapy varies accordingly (Arboix and Martí‐Vilalta 2004). In a patient with both Fabry disease and Bayés' syndrome, cerebrovascular risk may be compounded through two distinct mechanisms: small‐vessel cerebrovascular disease related to Gb3 accumulation in the cerebral vasculature, and cardioembolic risk related to atrial conduction disease and atrial fibrillation. This dual mechanism underscores the importance of recognizing Bayés' syndrome in this population, as it has direct implications for stroke prevention strategies.

Several limitations of this report should be acknowledged. First, as a single‐patient observation, this case cannot establish causality between Fabry disease and Bayés' syndrome; rather, it generates a hypothesis that warrants further investigation in larger cohorts. Second, although Gb3 accumulation in cardiomyocytes and conduction tissue has been well documented in Fabry disease, we did not have histologic confirmation of Bachmann's bundle involvement in this patient. Third, the patient's age (72 years) represents a potential confounder, as age‐related atrial fibrosis may have contributed to the electrophysiologic substrate for advanced interatrial block. Fourth, although cMRI demonstrated progressive ventricular LGE, atrial fibrosis could not be reliably assessed, limiting our ability to directly correlate imaging findings with the conduction abnormality. Finally, follow‐up was limited in duration, and longer‐term observation will be required to fully characterize the trajectory of her atrial conduction disease and arrhythmia burden, particularly with continued enzyme replacement therapy.

This case suggests several directions for future investigation. First, systematic ECG screening for interatrial block in patients with Fabry disease, both before and after initiation of enzyme replacement therapy, may help define the prevalence of atrial conduction disease in this population and clarify whether ERT modifies its natural history. Second, P‐wave‐centric ambulatory monitoring may be an important adjunct to standard 12‐lead ECG screening in this population, as advanced IAB physiology may be present despite a surface ECG that meets criteria only for partial IAB. Third, similar screening in other infiltrative cardiomyopathies, including cardiac amyloidosis, sarcoidosis, hemochromatosis, Pompe disease, and Danon disease, may identify whether Bayés' syndrome is a generalizable feature of infiltrative atrial cardiomyopathy or is more specific to particular disease mechanisms. Fourth, prospective studies are needed to determine whether early anticoagulation in patients with advanced interatrial block, prior to documented atrial fibrillation, reduces the incidence of cardioembolic events. Finally, advances in atrial‐specific cardiac imaging, including high‐resolution LGE protocols and atrial strain analysis, may improve our ability to noninvasively characterize the atrial substrate underlying Bayés' syndrome in patients with Fabry disease and other infiltrative cardiomyopathies.

## 4 Conclusion

Fabry disease is a rare X‐linked lysosomal storage disorder that can lead to multi‐organ dysfunction, including electrical conduction disease in the heart. To our knowledge this is the first known report identifying a potential connection between Fabry disease and Bayés' syndrome. In addition to considering advanced IAB and Bayés' syndrome as a consequence of Fabry disease, providers need to be able to recognize the ECG findings of advanced IAB and understand the elevated risk of developing atrial arrhythmias that it entails. Furthermore, this case highlights that advanced IAB physiology may not be apparent on the surface 12‐lead ECG and may require a P‐wave‐centric monitoring vector to be unmasked, with implications for diagnostic strategy in at‐risk populations.

## Author Contributions

**Nicholas E. Kunce:** conception, interpretation of data, drafting, and revising. **Andre Silva:** substantial drafting and revising. **Mark Haigney:** conception, interpretation of data, and revising. **Adam Kisling:** conception, interpretation of data, drafting, and revising. All authors have approved the submitted version of this manuscript.

## Funding

Partial support was received from award HU00012120008 from the Defense Health Agency to the Military Cardiovascular Outcomes Research program, Uniformed Services University, Bethesda, MD.

## Consent

Written informed consent for publication of their clinical details and/or clinical images was obtained from the patient in line with COPE guidelines.

## Conflicts of Interest

The authors declare no conflicts of interest.

## Data Availability

Data are available upon reasonable request to the corresponding author.
